# Using Thermal Signature to Evaluate Heat Stress Levels in Laying Hens with a Machine-Learning-Based Classifier

**DOI:** 10.3390/ani14131996

**Published:** 2024-07-06

**Authors:** Isaac Lembi Solis, Fernanda Paes de Oliveira-Boreli, Rafael Vieira de Sousa, Luciane Silva Martello, Danilo Florentino Pereira

**Affiliations:** 1 Business Administration Undergraduate, School of Sciences and Engineering, São Paulo State University (UNESP), Tupã 17602-496, SP, Brazil; isaac.l.solis@unesp.br; 2 Graduate Program in Agribusiness and Development, School of Sciences and Engineering, São Paulo State University (UNESP), Tupã 17602-496, SP, Brazil; ferzoovet@gmail.com; 3 Faculty of Animal Science and Food Engineering (FZEA), Department of Biosystems Engineering, University of São Paulo (USP), Pirassununga 13635-900, SP, Brazil; rafael.sousa@usp.br (R.V.d.S.); martello@usp.br (L.S.M.); 4 School of Sciences and Engineering, Department of Management, Development and Technology, São Paulo State University (UNESP), Tupã 17602-496, SP, Brazil

**Keywords:** featherless surface temperature, animal welfare, infrared thermography, supervised learning, data mining

## Abstract

**Simple Summary:**

This study proposes the use of the thermal signature method as a feature extractor from the temperature matrix obtained from regions of the body surface of laying hens (Gallus gallus domesticus) to enable the construction of a computational model for heat stress level classification. Rectal temperature was used to label each infrared thermography data as “Danger” or “Normal”, and five different classifier models (Random Forest, Random Tree, Multilayer Perceptron, K-Nearest Neighbors, and Logistic Regression) of rectal temperature class were generated using the respective thermal signatures. The Random Forest model for the face area of the laying hen achieved the highest performance (89.0%). For the wattle area, a Random Forest model also demonstrated high performance (88.3%), indicating the significance of this area in strains where it is more developed. These findings validate the method of extracting characteristics from infrared thermography.

**Abstract:**

Infrared thermography has been investigated in recent studies to monitor body surface temperature and correlate it with animal welfare and performance factors. In this context, this study proposes the use of the thermal signature method as a feature extractor from the temperature matrix obtained from regions of the body surface of laying hens (face, eye, wattle, comb, leg, and foot) to enable the construction of a computational model for heat stress level classification. In an experiment conducted in climate-controlled chambers, 192 laying hens, 34 weeks old, from two different strains (Dekalb White and Dekalb Brown) were divided into groups and housed under conditions of heat stress (35 °C and 60% humidity) and thermal comfort (26 °C and 60% humidity). Weekly, individual thermal images of the hens were collected using a thermographic camera, along with their respective rectal temperatures. Surface temperatures of the six featherless image areas of the hens’ bodies were cut out. Rectal temperature was used to label each infrared thermography data as “Danger” or “Normal”, and five different classifier models (Random Forest, Random Tree, Multilayer Perceptron, K-Nearest Neighbors, and Logistic Regression) for rectal temperature class were generated using the respective thermal signatures. No differences between the strains were observed in the thermal signature of surface temperature and rectal temperature. It was evidenced that the rectal temperature and the thermal signature express heat stress and comfort conditions. The Random Forest model for the face area of the laying hen achieved the highest performance (89.0%). For the wattle area, a Random Forest model also demonstrated high performance (88.3%), indicating the significance of this area in strains where it is more developed. These findings validate the method of extracting characteristics from infrared thermography. When combined with machine learning, this method has proven promising for generating classifier models of thermal stress levels in laying hen production environments.

## 1. Introduction

Thermal stress directly affects the physiological responses of chickens, leading to oxidative stress and autophagic dysfunction in the liver region, resulting in hepatic damage [1,2]. Birds are homeothermic animals, and a significant portion of the energy provided by feed is expended on maintaining body temperature [1]. Stress induced by high temperatures results in loss of appetite and consequently reduces food intake, diminishing the supply of metabolizable energy for production [3]. In the thermoneutral zone, birds expend less metabolic energy to maintain homeothermy and maximize their production. According to Ribeiro et al. [4], the ideal thermoneutral zone for chickens ranges from 21 °C to 28 °C.

Climate changes have led to increased temperatures in specific regions, presenting challenges to laying hen production [5]. To mitigate the effects of heat stress in tropical climates, investments in thermal animal response monitoring are essential to enable the design of climate control equipment and optimize the costs of poultry farms [2,6]. Studies by Ribeiro et al., Saeed et al., and Iyasere et al. [4,7,8] have demonstrated that climate-controlled poultry houses in tropical regions experience lower productivity losses and reduced mortality rates compared to non-climate-controlled houses.

When birds are subjected to stressful conditions, they can develop physiological responses through hormonal and hematological mechanisms aimed at preparing and providing necessary resources for the organism to cope with emergent compensatory demands, thereby maintaining bodily equilibrium. Examples of physiological responses include alterations in heart and respiratory rates, body temperature, and animal secretion [9]. Plumage and featherless regions play a significant role in avian thermoregulation [10]. Cândido et al. [11] demonstrated the relationship between internal body temperature and skin temperature in chickens, confirming that skin temperature can be used to estimate internal body temperature, serving as an indicator of thermal stress. Sensible heat loss decreases with increasing air temperature [3].

In chickens, sensible heat loss occurs through featherless body parts, such as the face and leg regions. Souza-Júnior et al. [12] used thermal images of chickens subjected to experiments in different thermal environment conditions and found that areas of the neck and featherless face exhibited higher values of sensible heat loss compared to the legs. Souza-Júnior et al., Zhao et al., and Cook et al. [12,13,14] have demonstrated the feasibility of assessing chicken feather scores using thermal images, which serve as a tool for mapping body surface temperature, thereby facilitating estimations of sensible heat loss.

Infrared thermography (IRT) is among the technologies under investigation for use in continuous monitoring systems to monitor changes in body surface temperature related to the response of the animal’s organism, including thermal stress, in a non-invasive and indirect manner [15]. In another vein, research into computational methods using machine learning techniques has yielded promising results in generating predictive models for assessing thermal stress using non-invasive sensors. Machine learning algorithms are recommended when working with large amounts of data and when there is no obvious correlation between the variables in a dataset, which could be studied with conventional statistical tools. Brown-Brandl et al. [16] constructed and evaluated five models for predicting thermal stress in beef cattle of varying breeds, including statistical models, Fuzzy Logic (FL) inference systems, and an artificial neural network (ANN). Hernandez-Julio et al. [17] investigated techniques for modeling physiological responses (rectal temperature and respiratory frequency) in Holstein dairy cows, employing only climatic data of air temperature and humidity as inputs. Pacheco et al. [18] proposed and assessed stress classifiers for dairy cows (Holstein) based on ANN-generated models. These models offered improved estimations of thermal stress levels compared to traditional Thermal Heat Index (THI) models and ANN-based models developed by other researchers. Rodrigues et al. [19] recently introduced a novel IRT data analysis method termed Thermal Signature. The authors’ method involves evaluating the frequency distribution of temperature pixels within a specific area of the thermal matrix. For cattle, the optimal region for extracting this thermal signature was the eyes. However, for chickens, it is necessary to determine whether the method can be applied and to identify the most suitable body part for analysis.

In this context, the hypothesis of this study is that there is a relationship between the temperature of featherless body surfaces and the internal temperature of laying hens that can be expressed through a body surface thermal profile, indicating comfort or stress conditions. This study proposes the application of the method called “Thermal Signature” for extracting features from data obtained by IRT and its use as an input attribute for machine-learning-based models to assess individual thermal stress conditions in laying hens.

## 2. Materials and Methods

The experiment was conducted in compliance with the guidelines set forth by UNESP’s Ethics Committee on Animal Use, under protocol no. 02/2022. The physiological and infrared thermography (IRT) data collected were utilized to develop the methodology referred to in the study as Thermal Signature (TS). Subsequently, the TS data were employed to test five machine learning methods (Random Forest, Random Tree, Multilayer Perceptron, K-Nearest Neighbors, and Logistic Regression) aimed at generating models that classify the animal’s thermal stress level.

### 2.1. Experimental Design and Data Collection

The research was conducted in two climate chambers. Both chambers have the same dimensions: 4.10 m in length, 3.50 m in width, and a ceiling height that varies from 2.85 to 3.15 m. A total of 192 laying hens from two strains (Dekalb White and Dekalb Brown), both 34 weeks old at the beginning of the experiment, were housed. Laying hens were not selected to ensure similar body weights. At the beginning of the experiment, the body weights of the Dekalb White birds were 1.59 ± 0.11 kg, and those of the Dekalb Brown birds were 1.92 ± 0.11 kg. The experiment was divided into two repetitions, each with 48 chickens from each strain. In the first repetition, one climate chamber was programmed to simulate a thermally comfortable environment, with temperatures of 26 °C during the day and 23 °C at night, while the other was programmed to simulate a thermally stressful environment, with temperatures of 35 °C during the day and 26 °C at night. The treatment application period was considered from 9 a.m. to 8 p.m. In both climate chambers, the relative air humidity was maintained at 60%. In the second stage, the same treatments were reversed between the climate chambers to control for room effects on the experimental data (45 weeks). The total duration of the experiment, including the sanitary void, was 16 weeks.

Within the chambers, the hens were housed in 12 cages, with six cages dedicated to each strain. The allocation of strains in the cages was determined by random selection. Each cage housed four chickens of the same strain. Feed was provided twice a day, with a daily quantity of 110 g per bird, in the same composition provided at the farm (2846 kcal/kg of metabolizable energy, 14.93% of crude protein, 4.48% of calcium, and 0.29% of available phosphorus), and water was provided ad libitum.

On the first day of housing, all hens were weighed on a platform scale (B-160, Líder Co., Ltd., Araçatuba, São Paulo, Brazil). One bird from each cage was randomly selected for weekly monitoring of blood parameters, rectal temperature, and weight, consistently from the same bird. The laying hens were handled inside the climatic chambers, between late morning and early afternoon. A handheld Digital Thermometer G-Tech^®^ (model TH1027, Joytech HealthCare Co., Zhejiang, China) with an accuracy of ±0.1 °C was inserted into the hen’s cloaca for rectal temperature measurement. These monitored birds were used to associate surface temperatures with internal temperature (rectal temperature).

IRT data from birds were captured weekly during the weighing process using the thermal camera Testo 880 (Testo SE & Co. KGaA, Darmstadt, Germany). Two thermal images (IRT data) were captured from a distance of 1 m for each chicken, one facing the camera and the other from the side. A total of 1920 thermal images were collected for processing and analysis, with 480 images (240 in each position: lateral and frontal) of the hens selected for weekly internal temperature measurement. All IRT data were automatically identified by the camera during registration and subsequently matched with laying hen identification. Environmental thermal conditions, bird identification, date of collection, and rectal temperature were associated with each thermal image to compose the dataset.

### 2.2. Data Organization and Pre-Processing

Chickens have most of their bodies covered with feathers, causing the surface temperature to be very close to the ambient air temperature. Therefore, there are few areas available to extract skin temperature and, consequently, obtain a TS. Six body areas were evaluated in this research: face, eye, wattle, comb, leg, and foot.

**Figure 1:**
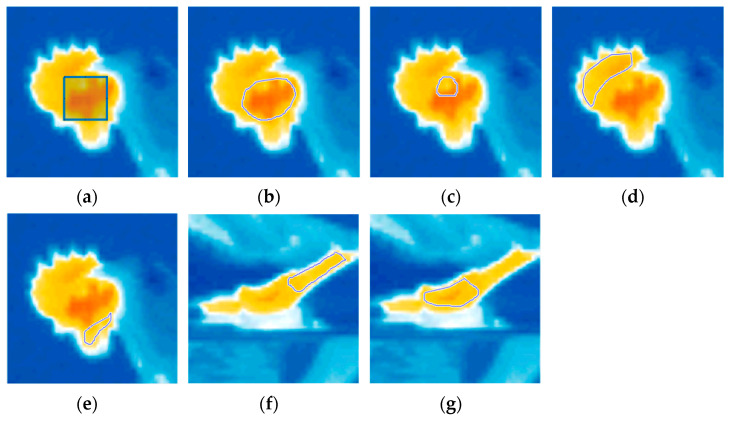
Selected body areas of the bird ((**a**,**b**)—face, (**c**)—eye, (**d**)—comb, (**e**)—wattle, (**f**)—leg, and (**g**)—foot) and the type of selection used ((**a**)—fixed-size rectangle, (**b**–**g**)—variable-size freehand).

Using the IRSoft 4.4 software from the thermographic camera (Testo SE & Co. KgaA, Germany), the temperature matrix of each evaluated image was exported. The temperature matrix corresponds to the surface temperature at each pixel of the thermographic image. In this study, each temperature matrix has a size of 120 × 160 pixels. A script in MATLAB R2021b (MathWorks Inc., Natick, MA, USA) and a graphical interface were developed to manually select the areas of interest on the chickens and correlate them with the temperature matrices, referred to as the Chicken Thermal Signature. The face region was evaluated using two types of selections: a fixed-size rectangular cutout (approximately 10% of the image width, in pixels) and a freehand selection. The other body areas were all obtained by freehand selection. Figure 1 shows examples of the selection types for these areas.

**Figure 2:**
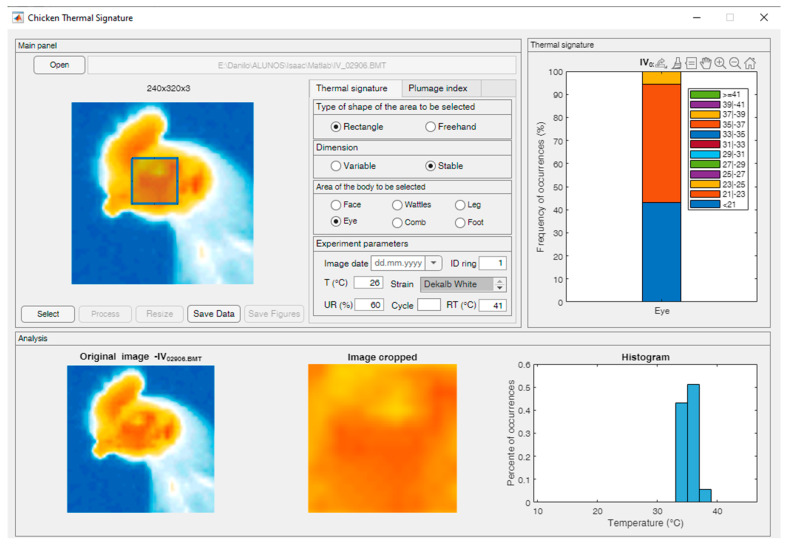
The screen of the Chicken Thermal Signature software showing the original thermal image, the selection of the eye region, the temperature histogram of this region, and the TS descriptor vector (graph).

The Chicken Thermal Signature software (version 1.0) provides tools to facilitate the selection of various areas of interest on the laying hens’ bodies, extract the corresponding area from the temperature matrix, and generate a labeled IRT database (RGB and TS). Figure 2 shows the interface of the Chicken Thermal Signature software.

To obtain the TS from IRT data, the software implements the three steps proposed by Rodrigues et al. [19], as summarized below:

1. Organization of the Temperature Matrix Database: each thermal image is downloaded and saved as a temperature matrix in ‘csv’ format using specialized software. A database of cutouts is built by manually selecting the regions of interest in RGB images corresponding to each temperature matrix. Sub-matrices of specified dimensions are generated.
2. Definition of Temperature Ranges: the number of cells in all temperature matrices is summed, and these temperatures are distributed into ranges. A balanced representation is ensured by grouping similar numbers of temperature cells per range. This process is automatically executed by a script, generating asymmetric range values to achieve homogeneity.
3. Calculation and Recording of Percentages: the percentage distribution of temperatures in each matrix is automatically counted. A descriptor vector is generated for each temperature matrix, detailing the percentage of temperature cells in each defined range.

The resulting TS is a descriptor vector for each IRT data, containing the percentage of temperatures within each range. The process is akin to creating a digital image histogram but tailored to operate with temperature matrices and specific temperature bands.

For each body region and selection method, the proportion of pixels observed within the selected area was divided into 12 temperature bands and saved: band 1: T < 21 °C, band 2: 21 °C ≤ T < 23 °C, band 3: 23 °C ≤ T < 25 °C, band 4: 25 °C ≤ T < 27 °C, band 5: 27 °C ≤ T < 29 °C, band 6: 29 °C ≤ T < 31 °C, band 7: 31 °C ≤ T < 33 °C, band 8: 33 °C ≤ T < 35 °C, band 9: 35 °C ≤ T < 37 °C, band 10: 37 °C ≤ T < 39 °C, band 11: 39 °C ≤ T < 41 °C, and band 12: T ≥ 41 °C. The data were saved in a spreadsheet and later used for training and validating computational models to classify thermal stress using rectal temperature as the gold standard.

### 2.3. Modelling Based on Supervised Learning

For constructing classifier models, the initial step involves labeling the data. This ensures that once the data is labeled into heat stress classes, it becomes usable for training the models using machine learning algorithms. The predictor attributes used for the models were the bands of the vector descriptor, as described in the previous section. The target attribute used was rectal temperature classes (T_R_), which were labeled into two distinct classes: ‘normal’ for T_R_ ≤ 41.0 °C, and ‘danger’ for T_R_ > 41.0 °C.

The dataset underwent training and tuning processes to identify the optimal combination of hyperparameters for machine learning algorithms, utilizing the Orange Data Mining 3.37.0 software (University of Ljubljana, Slovenia). The evaluated models comprised Random Tree (RT), Random Forest (RF), Logistic Regression (LR), K-Nearest Neighbors (KNN), and Multilayer Perceptron (MLP) algorithms. Tuning involves defining a range of potential values for each hyperparameter requiring adjustment. The algorithm then explores all feasible combinations of these values to assess the model’s performance across various configurations. The tuning process utilized the k-fold cross-validation technique with a value of K set to 10. Cross-validation serves as a critical method for evaluating the robustness and generalizability of machine learning models. It helps mitigate issues such as overfitting, wherein a model excessively tailors to the training data, resulting in poor performance on new, unseen data.

The selection of the algorithms is justified due to their widespread use in academic research, demonstrating consistently good results across various domains. These algorithms are commonly chosen because they offer diverse and robust methodologies for tackling classification and regression problems. Each algorithm has a unique structure and approach [20]:

- **Random Tree (RT)**: based on Decision Tree, it establishes rules for decision-making through a hierarchical structure where conditions at each node lead to different branches until a terminal node (leaf) is reached. The training algorithm seeks optimal conditions for this structure. In prediction problems, results are obtained by averaging the predicted values of each tree in the forest.
- **Random Forest (RF)**: this algorithm generates and aggregates the responses of multiple Random Trees created through supervised training. Combining multiple decision trees reduces the risk of overfitting and typically performs well in classification and regression problems. It is easy to interpret, with coefficients indicating the relationships between variables.
- **Logistic Regression (LR)**: used mainly for binary classification, it predicts the probability of an event belonging to one of two classes. Despite its name, it is used for classification tasks and can be extended to multiclass and ordinal regression problems. LR offers a straightforward probabilistic framework suitable for binary and multiclass classification.
- **K-Nearest Neighbors (KNN)**: this algorithm measures the similarity of a new observation to the k instances in a training set. It does not explicitly learn a model but uses training instances for prediction. The nearest k points are used to calculate class probabilities, assigning the observation to the class with the highest probability.
- **Multilayer Perceptron (MLP)**: uses the backpropagation algorithm to train models with a Multilayer Perceptron architecture. An MLP consists of an input layer, an output layer, and multiple hidden layers, allowing it to model complex, non-linear relationships. Training involves several hyperparameters, enabling flexibility and adjustability in supervised learning.

The performance of the trained models was assessed using metrics derived from confusion matrices and the Area Under the Receiver Operating Characteristic Curve (AUC-ROC) for binary classification [21]. Confusion matrices enable the evaluation of classifier efficiency in terms of accuracy, precision, recall, and F1 score. Although accuracy is an essential metric, it should not be considered in isolation due to its susceptibility to inaccuracies, especially in cases of unbalanced classes. Precision measures the classifier’s ability to avoid incorrect classifications, while recall reflects its ability to capture all correct samples for each class. Low precision indicates misclassification from other classes into the observed class, while low recall suggests difficulty in capturing correct examples. The F1 score, calculated as the harmonic mean of precision and recall, provides a balanced assessment of the classifier’s performance. All metrics are derived from the confusion matrix values: true positives (TP), true negatives (TN), false positives (FP), and false negatives (FN). TP and TN denote correct identifications, while FP and FN represent misclassifications.

(1) $$Accuracy=\frac{TP+TN}{TP+FN+FP+TN}$$

(2) $$Precision=\frac{TP}{TP+FP}$$

(3) $$Recall=\frac{TP}{TP+FN}$$

(4) $$F1\;Score=\frac{\;2\times Precision\times Recall}{Precision+Recall}$$

The ROC curve provides a graphical representation of a binary classification model’s performance across various discrimination thresholds, plotting the TP rate against the FP rate. A higher AUC-ROC value indicates better discrimination ability, with a maximum value of 1 representing perfect classification and a minimum value of 0.5 representing random classification.

(5) $$AUC=0.5*(\frac{\;TP}{TP+FN}+\frac{\;TN}{TN+FP})$$

## 3. Results

### 3.1. Rectal Temperatures

Based on the experimental data, it was examined whether there is a difference in the rectal temperatures of hens subjected to stress treatments compared to those in the comfort group, and between different breeds. Table 1 shows the confidence interval plots for the Dekalb White and Dekalb Brown breeds, displaying the observed mean rectal temperature for each temperature treatment.

For both breeds, an increase in rectal temperature was observed in birds subjected to thermal stress. ANOVA indicated no significant difference in rectal temperature between the breeds, but there was a significant difference between the treatments (*p* < 0.01).

### 3.2. Infrared Thermography Datasets

During the preprocessing stage, not all thermal images provided adequate information for the areas of the body surface of interest described earlier. Depending on the laying hen’s position in the captured image, some parts of the body were not adequately exposed, and as a result, these areas were not delineated and did not provide information for the classification models. Table 2 summarizes the size of the datasets generated for each body part and type of selection.

The records classified as “Danger” and “Normal” in the Infrared thermography datasets were unbalanced. To address the class imbalance and provide more robust and balanced machine learning models, the supervised filters Resample and Class Balancer were applied. These filters help mitigate classifier bias towards the majority class, promoting a fairer and more accurate analysis of minority classes. The Resample filter is used to create a subset of samples from the original dataset, and the Class Balancer filter adjusts the class distribution in the dataset to enhance classifier performance.

### 3.3. Computational Models

From the balanced datasets, we generated computational classification models (thermal stress level classifiers) and compared their performances. Table 3 presents the performance metrics for the computational model generated from the predictor attribute set consisting of the TS of all regions of interest without differentiation of breed, body part, or selection type.

As observed in Table 3, the computational model generated using the Random Forest method exhibited better performance, with AUC, accuracy, and F1 score equal to 89.0%, 83.4%, and 83.4% respectively. However, this model utilizes all areas of interest, which can be challenging for a computer vision system with automatic identification and cropping of these regions. In order to simplify the classification models from the perspective of this difficulty, computational classifier models were generated for each TS of each individual region of interest. Table 4 shows the results of the comparison metrics for each model obtained with the Identification Datasets described in Table 2.

As observed in Table 4, Random Forest was also the best machine learning tool for generating the thermal stress level classifier models for the different datasets. The classifiers with the best performance were those generated with Random Forest for the dataset with TS extracted from the face using fixed-size rectangle selection type (Dataset I), and with TS extracted from the wattles using variable-size freehand selection type (Dataset V). The classifier for the face region achieved a performance with AUC, accuracy, and F1 score equal to 89.0%, 83.4%, and 83.4% respectively. The classifier for the wattles region achieved a performance with AUC, accuracy, and F1 score equal to 88.3%, 83.6%, and 83.6%, respectively.

Although the classifiers using the TS of the face and wattles showed similar performance, applying the former in a computer vision system would be simpler as it uses a fixed-size geometric shape (fixed-size rectangle) for the cropping mechanism in the specified region of the hen’s body surface. Table 5 details the performance of the Random Forest classifier generated with the TS from the face region using fixed-size rectangle selection.

As shown in Table 5, the classifier using the facial TS demonstrates similar accuracy across classes. However, the precision and recall (83.9% and 85.9% respectively) for the ‘Danger’ class are slightly better. This is a positive outcome, as misclassification errors in this class could lead to more significant issues in the production system with laying hens.

## 4. Discussion

It was observed that the rectal temperature of chickens increased under heat stress by approximately 0.2 °C. Cândido et al. [11] obtained similar results, where at 35.0 °C, the average rectal temperature observed was 42.9 °C. Kim et al. [22] found that rectal temperature, surface temperature, and heart rate were significantly higher in birds subjected to thermal stress. Abreu et al. [23] also noted that rectal temperature increased by approximately 0.4 °C when the birds were subjected to thermal stress. The results found expose the susceptibility of laying hens to heat stress and the importance of recognizing its effects early in order to minimize losses.

The analysis of body surface temperature in birds and its association with internal body temperature is a different challenge compared to fur-bearing animals. The entire area covered with feathers correlates more with ambient temperature than body temperature. Few exposed skin areas favor heat exchange between the animal and the environment. As verified in this research, not all areas of the body are suitable for making this association between skin temperature and core temperature. The wattles and faces were better for estimating an increase in internal temperature when compared to combs, legs, and feet.

The method of summarizing surface temperature information from body parts of laying hens into a Thermal Signature has been validated. There are no records of approaches similar to the one proposed in this study for poultry, as they are more commonly found for beef and dairy cattle. Pacheco et al., Rodrigues et al., and Pacheco et al. [18,20,24] developed models to relate surface temperature with rectal temperature and other physiological parameters in cows, achieving the best performance with artificial neural network-based models. Rodrigues et al. [19] achieved better explanatory power in artificial neural network models that related surface temperature to respiratory rate using infrared thermography data of the forehead, with an accuracy of 80.8%, and to rectal temperature using infrared thermography data of the eyes, with an accuracy of 90.1%. In this research, the best results were obtained from Random Forest models, with accuracies above 88% for models based on wattles and faces.

In comparison, Pacheco et al. [18] employed infrared thermography data as input for artificial neural networks to predict heat stress levels in dairy cows based on rectal temperature values, achieving a maximum accuracy of 84.0%. The similar accuracy achieved in our study can be attributed to the greater robustness of the thermal signature methodology used to extract features from infrared thermography. This method considers all areas of interest within the images rather than specific points, thus making it more robust against measurement uncertainties. Additionally, Pacheco et al. [24] utilized infrared thermography as input for convolutional neural networks to develop models for predicting heat stress levels in dairy cows. The classification was based on ranges of rectal temperature and respiratory rate. The highest accuracy achieved by the model using rectal temperature values was 70.5%, while the model based on respiratory rate achieved a maximum accuracy of 76.3%.

Obtaining models that correctly classify chickens with increased internal body temperature, based on surface temperature, and with accuracies of approximately 90%, makes monitoring birds using thermal imaging potentially advantageous for the poultry industry. New studies must be conducted, associating the Thermal Signature with other physiological variables, such as respiratory rate and chicken pathologies. Improving this methodological approach will allow the technological development of tools to help monitor commercial farms in the future for earlier diagnosis and intervention.

## 5. Conclusions

The study presented a method for developing machine learning models to predict the thermal stress levels of laying hens using infrared thermography data from featherless regions of the body surface. No differences were observed in the thermal signature of surface temperature and rectal temperature between the strains. It was evidenced that rectal temperature and the thermal signature express the conditions of heat stress and comfort. The Random Forest model for the face area of the laying hen achieved the highest performance. For the wattle area, a Random Forest model also demonstrated high performance, indicating the significance of this area in strains where it is more developed. The thermal signature proved to be an effective predictive attribute for the models using infrared thermography data extracted from the body surface of laying hens. When combined with machine learning, this method has proven promising for generating classifier models of thermal stress levels in laying hen production environments.

## Figures and Tables

**Table 1:** Mean and standard error of rectal temperature (°C) measured throughout the experimental period in laying hens of the Dekalb White and Dekalb Brown genetics.

Temperature Treatment	Dekalb White	Dekalb Brown
26 °C	40.9 ± 0.04	41.2 ± 0.06
35 °C	40.8 ± 0.07	41.2 ± 0.07

**Table 2:** Characterization of the infrared thermography datasets generated for each region of the body surface and type of selection.

Dataset Identification	Body Surface	Selection Type	Dataset Size	Description
I	Face	Fixed-size rectangle	158	Area of the head encompassing the eye, ear, and earlobe
II		Variable-size freehand	233	
III	Eye	Variable-size freehand	234	Region surrounding the eye
IV	Comb	Variable-size freehand	220	Area of the comb, excluding the tips
V	Wattle	Variable-size freehand	190	Area of the wattles
VI	Leg	Variable-size freehand	151	Area below the feathered part down to the meeting point of the leg
VII	Foot	Variable-size freehand	162	Exposed area of the foot in the image, excluding the toes

**Table 3:** Performance of the classification models generated for the thermal signatures of all body areas in the dataset (all data).

Classifier Type	AUC *	Accuracy	F1 Score
Random Forest	89.0%	83.4%	83.4%
Random Tree	82.1%	74.5%	74.6%
Multilayer Perceptron	82.2%	72.0%	71.7%
K-Nearest Neighbors	79.0%	68.8%	68.8%
Logistic Regression	67.0%	61.1%	60.9%

* AUC: Area Under the Receiver Operating Characteristic Curve.

**Table 4:** Performance of the classification models generated for each thermal signature related to each body surface area dataset.

Dataset Identification	Classifier Type	AUC *	Accuracy	F1 Score
I	Random Forest	89.0%	83.4%	83.4%
	Random Tree	82.1%	74.5%	74.6%
	Multilayer Perceptron	82.2%	72.0%	71.7%
	K-Nearest Neighbors	79.0%	68.8%	68.8%
	Logistic Regression	67.0%	61.1%	60.9%
II	Random Forest	87.1%	75.9%	79.4%
	Random Tree	76.7%	71.0%	70.6%
	Multilayer Perceptron	71.7%	68.8%	68.7%
	K-Nearest Neighbors	73.6%	69.2%	69.2%
	Logistic Regression	68.4%	71.0%	70.7%
III	Random Forest	88.0%	82.2%	82.2%
	Random Tree	75.3%	66.4%	66.2%
	Multilayer Perceptron	66.8%	64.7%	64.5%
	K-Nearest Neighbors	78.1%	71.8%	71.8%
	Logistic Regression	56.7%	58.9%	58.3%
IV	Random Forest	83.0%	76.5%	76.5%
	Random Tree	74.0%	73.0%	72.9%
	Multilayer Perceptron	70.1%	69.9%	69.8%
	K-Nearest Neighbors	67.2%	60.7%	60.6%
	Logistic Regression	68.4%	68.9%	68.9%
V	Random Forest	88.3%	83.6%	83.6%
	Random Tree	71.2%	67.3%	67.0%
	Multilayer Perceptron	70.6%	70.0%	69.8%
	K-Nearest Neighbors	76.8%	69.1%	69.1%
	Logistic Regression	64.7%	65.0%	65.1%
VI	Random Forest	86.1%	79.5%	79.1%
	Random Tree	71.7%	62.3%	61.6%
	Multilayer Perceptron	72.7%	71.9%	68.9%
	K-Nearest Neighbors	77.5%	73.3%	73.3%
	Logistic Regression	60.2%	61.0%	59.5%
VII	Random Forest	88.0%	80.5%	80.4%
	Random Tree	78.2%	73.2%	73.2%
	Multilayer Perceptron	76.7%	72.6%	72.0%
	K-Nearest Neighbors	71.0%	62.2%	62.2%
	Logistic Regression	62.8%	59.1%	58.9%

* AUC: Area Under the Receiver Operating Characteristic Curve.

**Table 5:** Confusion matrix for the classifier built using Random Forest for thermal signatures extracted from the hen’s face.

Observed Class	Predicted		Recall (%)
	Normal	Danger	
Normal	58	14	80.6
Danger	12	73	85.9
Precision (%)	82.9	83.9	Accuracy 83.4%

## Data Availability

Data will be made available upon request to the corresponding author.
